# Effects of adjunctive milrinone versus placebo on hemodynamics in patients with septic shock: a randomized controlled trial

**DOI:** 10.1080/07853890.2025.2484464

**Published:** 2025-03-26

**Authors:** Surat Tongyoo, Suratee Chobngam, Nutnicha Yolsiriwat, Chutima Jiranakorn

**Affiliations:** a Faculty of Medicine, Siriraj Hospital, Mahidol University, Bangkok, Thailand; b Internal Medicine, Hatyai Hospital, Hatyai, Songkla, Thailand

**Keywords:** Inotrope, milrinone, septic shock, sepsis induced cardiomyopathy, phosphodiesterase inhibitor, resuscitation

## Abstract

**Background:**

Refractory septic shock can lead to multiorgan failure and death due to myocardial dysfunction-induced inadequate tissue perfusion. Current guidelines advocate inotropic adjuncts to norepinephrine, but the efficacy of milrinone remains understudied in this context. This study aimed to evaluate the hemodynamic changes in septic shock patients treated with adjunctive milrinone compared to those treated with a placebo.

**Methods:**

This multicenter, double-blind, randomized controlled trial enrolled adults with septic shock, adequate fluid resuscitation, and a mean arterial pressure ≥ 65 mmHg. Eligible patients exhibited poor tissue perfusion or impaired left ventricular systolic function. Participants were randomized 1:1 to milrinone or placebo. Echocardiographic hemodynamic assessments were performed pre- and postintervention. The primary outcome was the change in cardiac output from baseline to 6 h after drug administration. The study was prospectively registered at www.clinicaltrials.gov (NCT 05122884).

**Results:**

Among 271 screened patients, 64 were randomized. The baseline characteristics were comparable between the groups. The milrinone group demonstrated a significantly greater change in cardiac output at 6 h (median [IQR] 0.62 L/min [-0.51 to 1.47]) than did the placebo group (0.13 L/min [-0.59 to 0.46]; *p* = 0.043). The percentage change in the cardiac index was also significantly greater with milrinone (median [IQR] 22.5% [-10.4% to 45.3%]) than with placebo (4.4% [-10.9% to 11.4%]; *p* = 0.041). There were no significant differences in complication rates between the groups. The 28-day mortality rates of the groups were also statistically nonsignificant and equivalent (16/32 [50.0%] for both; *p* = 1.000).

**Conclusions:**

Milrinone administration in septic shock patients improved cardiac output at 6 h, suggesting a potential benefit for patients with persistent tissue hypoperfusion despite norepinephrine.

## Introduction

Septic shock, which is characterized by high mortality and substantial economic burden, remains a critical global health concern. The World Health Organization reported approximately 30 million annual sepsis cases worldwide in 2016, resulting in up to 6 million deaths [1]. In Asia, septic shock mortality rates reach 44.2%, with Thailand reporting rates as high as 59% [2,3]. Refractory shock and multiorgan failure are the primary causes of death in these patients [4,5].

Myocardial dysfunction frequently complicates septic shock [6], leading to decreased stroke volume and cardiac output. This compromised circulation results in inadequate tissue perfusion, multiorgan failure, and increased mortality. Recent studies have consistently correlated myocardial dysfunction in sepsis or septic shock with increased mortality [7,8].

Current treatment guidelines include the Surviving Sepsis Campaign Guideline 2021 and the Japanese Clinical Practice Guidelines for Management of Sepsis and Septic Shock 2020. These guidelines recommend the addition of dobutamine to norepinephrine or epinephrine monotherapy for adults with septic shock if there is cardiac dysfunction and evidence of poor tissue perfusion, despite adequate volume status and blood pressure [9,10].

Despite its efficacy in augmenting cardiac output, dobutamine administration has been associated with increased mortality in several studies [11–13]. Furthermore, combining dobutamine, a synthetic β1-receptor agonist, with the more potent catecholamine norepinephrine may fail to improve cardiac output and potentially exacerbate tachyarrhythmias.

Milrinone, a phosphodiesterase type III inhibitor, increases intracellular cyclic adenosine monophosphate, acting as an inotropic agent with pulmonary vasodilatory effects [14]. Due to its distinct mechanism of action from that of sympathetic inotropes, milrinone is a promising option for enhancing cardiac function in sepsis patients *via* non-catecholaminergic pathways. However, the literature on the role of milrinone in septic shock treatment remains limited [15,16]. A recent retrospective study found that phosphodiesterase 3 inhibitor administration did not improve lactate clearance, organ failure resolution, or survival in septic shock patients with high lactate levels. Similarly, an analysis of the MIMIC III public database showed that milrinone did not improve hospital mortality compared to dobutamine in sepsis patients. However, a separate study on children with Enterovirus 71-induced brain stem encephalitis found that milrinone improved outcomes by reducing inflammation and stabilizing hemodynamics, suggesting its potential as a therapeutic option for this severe condition [17–19].

This study’s primary objective was to compare the efficacy of adjunctive milrinone versus that of placebo in enhancing cardiac output among adult septic shock patients. We aimed to assess this efficacy in patients who were resuscitated to target blood pressure but still exhibited poor tissue perfusion or myocardial dysfunction.

## Methods

### Study design and setting

We conducted a double-blind, randomized controlled trial at Siriraj Hospital, Mahidol University (a tertiary referral center in Bangkok, Thailand), and Hatyai Hospital (another tertiary referral center in southern Thailand). The protocol was approved by the Siriraj Institutional Review Board (certificate of approval no. Si 111/2021) and was conducted under the ethical principles of the Declaration of Helsinki. The Siriraj Research Development Fund (grant number: IO-R016431074) supported this research. The study was prospectively registered at www.clinicaltrials.gov (NCT 05122884) under the original title ‘Milrinone Versus Placebo in Patients With Septic Shock’, with a registration date of September 23, 2021. Although the title was modified following language editing, there were no deviations from the original trial protocol. The full study protocol has been published elsewhere [20]. The reporting of this study conforms to the CONSORT (Consolidated Standards of Reporting Trials) statement [21].

### Study participants

We screened adults aged ≥ 18 years with septic shock as defined by SEPSIS III [22]. Eligible patients were those who had received ≥ 30 mL/kg of fluid resuscitation and/or vasopressors to maintain a mean arterial pressure of ≥ 65 mmHg but still showed poor tissue perfusion or impaired left ventricular systolic function (left ventricular ejection fraction < 40%). Poor tissue perfusion was defined as persistent serum lactate > 2 mmol/L and/or urine output < 0.5 mL/kg at 6 h post-resuscitation. The exclusion criteria were as the following: no evidence of poor tissue perfusion and left ventricular ejection fraction >40%, life-threatening tachyarrhythmia pre-enrollment, chronic kidney disease stage 5 patients who refused renal replacement therapy, and terminally ill patients with do-not-resuscitate orders.

### Recruitment

Eligible patients or their family members were informed about the study process, and the written informed consent was obtained from participants or their legal guardians. Participants were randomly assigned at a 1:1 ratio through simple randomization based on sequential enrollment numbers. Allocation concealment was ensured using a computer-generated randomization table provided by an independent investigator. All other investigators, patients, attending physicians, and nurses remained blinded to the study assignments.

### Interventions

Patients who met the inclusion criteria received treatment according to septic shock management guidelines. The intervention group received 20 mg of intravenous milrinone in 100 mL of normal saline solution at 0.5 mg/kg/min for ≥ 12 h. The placebo group received 100 mL of normal saline solution prepared identically, with matching packaging, administration rate, and route. Additional interventions were determined based on individual patient conditions and physician judgment.

Physicians could adjust vasopressor dosages if the mean arterial pressure fell below 65 mmHg. Alternatively, if the mean exceeded ≥ 75 mmHg for > 30 min, vasopressor doses were reduced (epinephrine first, followed by norepinephrine). Other treatments, including fluid infusion, antimicrobial therapy, nutritional support, mechanical ventilation, and renal replacement therapy, were administered at the discretion of the attending physicians.

Investigators performed echocardiograms to assess cardiac function, including cardiac output, left ventricular ejection fraction, and inferior vena cava diameter variation, before drug administration and at 6 and 24 h after administration. The full echocardiogram protocol was described in previous publications [23,24]. To ensure data consistency, operators underwent a standardized transthoracic echocardiography workshop, with inter- and intra-observer variabilities maintained below 15% before data collection commenced. Vital signs, urine output, and serum lactate levels were monitored and recorded at 6 and 24 h after the commencement of milrinone or placebo.

### Outcome measurement

The primary outcome was cardiac output change from baseline to 6 h after drug administration. The secondary outcomes compared between the milrinone and placebo groups were the 28-day, ICU, and in-hospital mortality rates; serum lactate clearance; left ventricular ejection fraction change at 6 h; and the number of organ support-free days. Significant cardiac index improvement was defined as *a* > 15% increase, with proportions compared between the two study groups.

The safety outcomes included monitoring for new-onset cardiac arrhythmias and milrinone-associated complications. Physicians stopped the study drug if there were any suspected severe side effects (ventricular fibrillation, ventricular tachycardia, or unstable atrial arrhythmias) and promptly notified the research team. Stable arrhythmias, occasional premature ventricular contractions, or premature atrial contractions were managed per guidelines. Basic laboratory tests were performed to monitor organ function.

### Sample size calculation

The sample size was calculated based on observed cardiac output changed in a previous study, published in 2015 [17]. We hypothesized that the baseline cardiac output would be 3.0 ± 0.8 L/min, increasing to 3.6 ± 0.8 L/min in the milrinone group but remaining unchanged in the control group. With 5% type I error and 20% type II error, 28 patients per group were needed. After allowing for a 10% data loss, we determined that the total sample size should be 64 patients.

### Statistical analysis plan

Categorical data are presented as frequencies and percentages. Quantitative variables were tested for distribution using the Kolmogorov–Smirnov test and are reported as the means and standard deviations if normally distributed or as medians and interquartile ranges if not normally distributed. The chi-square test or Fisher’s exact test was used to compare categorical factors between groups. Independent samples t tests or Mann–Whitney U tests were used for continuous data, as appropriate. *P* values < 0.05 were considered to indicate statistical significance.

## Results

### Patient characteristics

**Figure 1.**
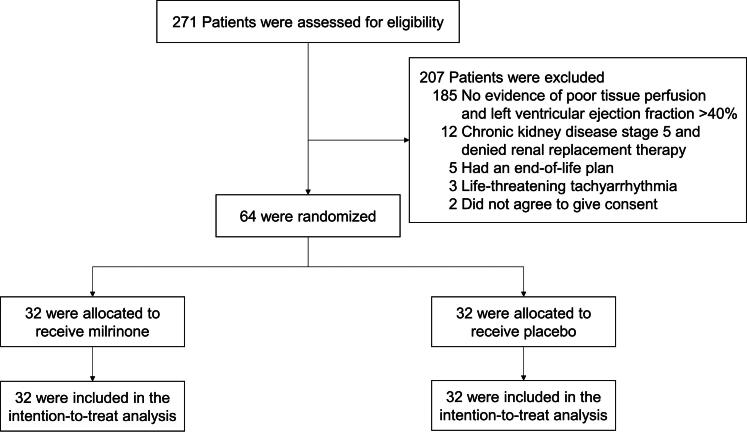
Patient flow diagram: screening, enrollment, and randomization process.

The study was conducted from 1^st^ December 2021 through 31^st^ March 2024, we screened 271 septic shock patients with clinical hypoperfusion or impaired left ventricular function in medical intensive care units at Siriraj and Hatyai Hospitals. After exclusion, 64 patients were randomized: 32 to the milrinone group and 32 to the placebo group (Figure 1). No patients withdrew, ensuring a complete final analysis.

The baseline characteristics were similar between the groups (Table 1). Comparisons are reported as milrinone vs placebo. No significant differences were observed in age, sex, body mass index, SOFA score, APACHE II score, or comorbidities. The primary infection sources were the respiratory tract, urinary tract, and septicemia. The baseline hemodynamics, including mean arterial pressure and heart rate, were comparable. The inferior vena cava diameter variation was similar between the groups.

**Table 1.** Baseline characteristics of the study participants.

Clinical parameters	Milrinone (*n* = 32)	Placebo (*n* = 32)	*P*
Age, median (IQR), y	68.5 (54-78)	73.5 (62-84)	0.270
Sex, *n* (% male)	15 (46.9)	19 (59.4)	0.316
Body mass index, median (IQR), kg/m^2^	22.3 (19.7-26.2)	22.9 (20.5-26.1)	0.672
APACHE II score, median (IQR)	18 (15-24)	21 (17-23.75)	0.310
SOFA score, median (IQR)	10.5 (10-12)	10.5 (8-13)	0.882
Underlying diseases, *n* (%)			
Hypertension	17 (53.1)	22 (68.8)	0.200
Diabetes mellitus	7 (21.9)	13 (40.6)	0.177
Chronic kidney disease	9 (28.1)	6 (18.8)	0.556
Coronary artery disease	5 (15.6)	6 (18.8)	1.000
Cerebrovascular disease	2 (6.3)	6 (18.8)	0.257
Atrial fibrillation	3 (4.4)	2 (6.3)	1.000
Site of infection, *n* (%)			
Respiratory tract infection	13 (40.6)	20 (62.5)	0.133
Urinary tract infection	6 (18.8)	7 (21.9)	1.000
Septicemia with unknown infected site	8 (25.0)	2 (6.3)	0.082
Intra-abdominal infection	4 (12.5)	2 (6.3)	0.672
Central nervous system infection	1 (3.1)	1 (3.1)	1.000
Vital signs and hemodynamic parameters, median (IQR)			
Temperature, °C	36.8 (36.0-37.8)	37.1 (36.5-37.9)	0.285
Mean arterial blood pressure, mmHg	81 (70-90)	75 (66-86)	0.168
Heart rate, beats per min	107 (96-121)	99 (80-114)	0.108
Inferior vena cava diameter variation, %	13.8 (6.7-20.9)	15.6 (9.3-25.1)	0.263
Serum lactate, mmol/L	4.3 (2.7-7.9)	3.4 (2.2-7.2)	0.273
Serum lactate > 2 mmol/L, *n* (%)	31 (96.9)	30 (93.8)	1.000
Left ventricular ejection fraction, %	44.8 (37.7-58.5)	55.9 (42.2-62.6)	0.199
Left ventricular ejection fraction <40%, *n* (%)	19 (59.4)	13 (40.6)	0.211
Cardiac output, L/min	3.5 (2.7-5.6)	4.2 (3.2-5.8)	0.151
Cardiac index, L/min/m^2^	2.1 (1.6-3.4)	2.5 (2.1-3.4)	0.179
Cardiac index < 2.2 L/min/m^2^	17 (53.1)	11 (34.4)	0.207
Fluid resuscitation, mL/kg	36.2 (22.9-58.5)	33.3 (21.9-40.4)	0.384
Vasoactive drugs, *n* (%)			
Norepinephrine alone	25 (78.1)	28 (87.5)	0.500
Norepinephrine plus adrenaline	2 (6.3)	1 (3.1)	0.788
Norepinephrine plus adrenaline plus dobutamine	3 (9.4)	1 (3.1)	0.672
Norepinephrine plus dobutamine	2 (6.3)	1 (3.1)	0.788
Dopamine plus adrenaline	0 (0)	1 (3.1)	1.000
Vasoactive dose, median (IQR), mcg/kg/min*	0.16 (0.07-0.26)	0.12 (0.03-0.27)	0.460
Hydrocortisone, *n* (%)	25 (78.1)	28 (87.5)	0.509
Renal replacement therapy, *n* (%)	11 (34.4)	7 (21.9)	0.410
Mechanical ventilation, *n* (%)	32 (100)	32 (100)	1.000

°C, degrees Celsius; APACHE II, Acute Physiology and Chronic Health Evaluation II; IQR, interquartile range; kg/m^2^, kilogram per square meter; L/min, liters per minute; L/min/m^2^, liters per minute per square meter; mcg/kg/min, micrograms per kilogram per minute; min, minute; mL/kg, milliliters per kilogram; mmHg, millimeters of mercury; mmol/L, millimole per liter; SOFA, sequential organ failure assessment; y, years.

^*^ Vasoactive dose or norepinephrine dose equivalents [30] were calculated with the following equation: Norepinephrine dose equivalent (micrograms per kilogram per minute [mcg/kg/min]) = (norepinephrine [mcg/kg/min] + epinephrine [mcg/kg/min] + dopamine [mcg/kg/min])/100.

Norepinephrine was the most frequently used vasoactive agent in this study. It was administered to all enrolled patients except for a single patient in the placebo group, who received a combination of dopamine and adrenaline. In the milrinone group, two patients received norepinephrine in combination with adrenaline, two received norepinephrine with dobutamine, and three received norepinephrine with both adrenaline and dobutamine. In the placebo group, one patient received norepinephrine with adrenaline, one received norepinephrine with dobutamine, and one received norepinephrine with both adrenaline and dobutamine. The median norepinephrine-equivalent dose for all vasoactive agents was 0.15 mcg/kg/min (IQR 0.07–0.26) in the milrinone group and 0.12 mcg/kg/min (IQR 0.03–0.27) in the placebo group, with no statistically significant difference between groups (*p* = 0.460) (Table 1).

No significant differences between the milrinone and placebo groups were found in baseline cardiac output, LV ejection fraction, or lactate levels. The fluid resuscitation volume, hydrocortisone use, renal replacement therapy, and mechanical ventilator use were similar (Table 1).

### Primary outcome

The change in cardiac output from baseline to 6 h was significantly greater in the milrinone group (0.62 L/min, IQR −0.51 to 1.47) than in the placebo group (0.13 L/min, IQR −0.59 to 0.46; *p* = 0.043; Table 2). Cardiac index changes were also significantly higher in the milrinone group, both in absolute value and percentage change (*p* = 0.039 and *p* = 0.041, respectively).

**Table 2.** Primary and secondary outcome measures.

Outcomes	Milrinone (*n* = 32)	Placebo (*n* = 32)	*P*
Primary outcomes			
Cardiac output change (baseline–6 h), median (IQR), L/min	0.62 (-0.51 to 1.47)	0.13 (-0.59 to 0.46)	0.043
Cardiac index change (baseline–6 h), median (IQR), L/min/m^2^	0.39 (-0.31 to 0.97)	0.08 (-0.30 to 0.29)	0.039
Cardiac index change, median (IQR), % change	22.5 (-10.4 to 45.3)	4.4 (-10.9 to 11.4)	0.041
**Secondary outcomes**			
28-d mortality, *n* (%)	16 (50)	16 (50)	1.000
Hospital mortality, *n* (%)	18 (56.3)	17 (53.1)	0.802
ICU mortality, *n* (%)	10 (31.3)	11 (34.4)	0.790
Cardiac index increasing > 15%	19/32 (59.4%)	5/32 (15.6%)	0.001
Left ventricular ejection fraction change^a^ (%)	6.1 (-6.5 to 22.3)	0.2 (-3.6 to 11.9)	0.274
Serum lactate at 24 hours, mmol/L	2.3 (1.6-4.0)	1.9 (1.6-3.6)	0.339
Serum lactate decrease^b^, %	41 (19-60)	40 (17-56)	0.877
ICU length of stay, median (IQR)	10 (5-20)	15 (8-21)	0.316
Hospital length of stay, median (IQR)	17 (9-39)	24 (17-33)	0.200
Ventilator free days in 28 days, median (IQR)	0 (0-9)	0 (0-19)	0.349
Vasopressor free days in 28 days, median (IQR)	12 (0-25)	13 (0-26)	0.351
RRT free day in 28 days, *n* (%)	0 (0-0)	0 (0-0)	0.508
Adverse events, *n* (%)			
Arrhythmia	4 (12.5)	4 (12.5)	1.000
- Atrial fibrillation	2 (6.3)	4 (12.5)	0.672
- Supraventricular tachycardia	2 (6.3)	0 (0)	0.492
Digital gangrene	1 (3.1)	1 (3.1)	1.000

d, days; h, hours; ICU, intensive care unit; IQR, interquartile range; L/min, liters per minute; L/min/m^2^, liters per minute per square meter; mmol/L, millimoles per liter.

^a^ Left ventricular ejection fraction (LVEF) change (%) was calculated with the following equation:.

([LVEF at 24 h – LVEF at baseline]/LVEF at baseline) × 100.

^b^ The decrease in the serum lactate concentration (%) was calculated with the following equation:.

([serum lactate at baseline – serum lactate at 24 h after start study drug]/serum lactate at baseline) × 100.

## Secondary outcomes

No significant differences were detected between the milrinone and placebo groups in terms of ICU mortality (31.3% vs 34.4%; *p* = 0.790), hospital mortality (56.3% vs 53.1%; *p* = 0.802), or 28-day mortality (50% vs 50%; *p* = 1.000). ICU and hospital lengths of stay and organ support-free days were similar between the groups (Table 2). Among patients with a cardiac index lower than 2.2 L/min/m^2^, the 28-day mortality rate was 52.9% in the milrinone group compared to 54.5% in the placebo group (*p* = 1.000). For those with a left ventricular ejection fraction lower than 40%, the 28-day mortality rate was 57.9% in the milrinone group versus 46.2% in the placebo group (*p* = 0.720) (Supplemental Table).

**Figure 2.**
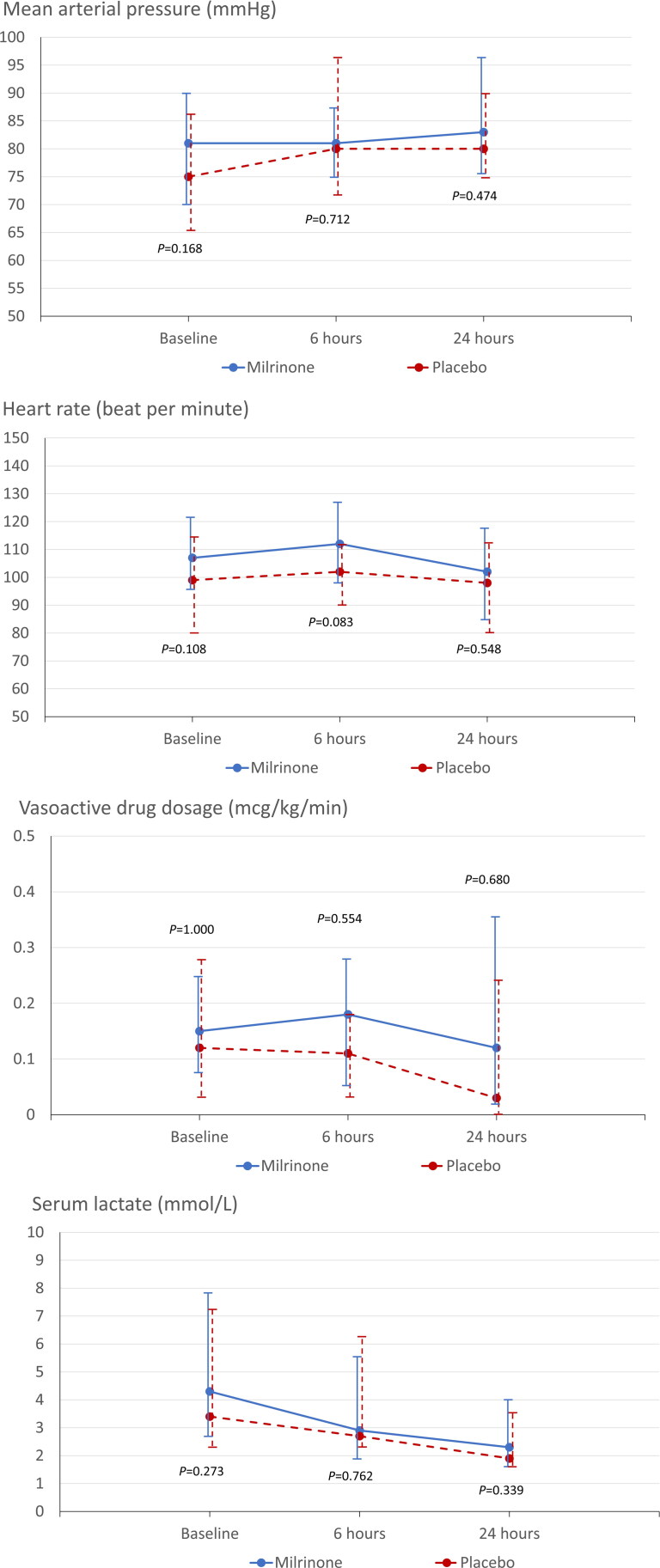
Comparison of the temporal trends in hemodynamic and metabolic parameters between the milrinone and placebo groups. (a) Mean arterial pressure; (b) heart rate; (c) vasoactive drug dosage; and (d) serum lactate.

The decrease in the serum lactate concentration was comparable between the milrinone and placebo groups (41% vs 40%; *p* = 0.877; Table 2). Figure 2 present trends in heart rate, mean arterial pressure, vasoactive drug dosage, and serum lactate level.

### Adverse events

Cardiac arrhythmias occurred in 4/32 patients (12.5%) in both groups. The milrinone group experienced two cases each of atrial fibrillation and supraventricular tachycardia, while all four arrhythmic events in the placebo group were atrial fibrillation. In addition, one case of digital gangrene was reported in each group.

### Subgroup analysis

A significant improvement in the cardiac index (> 15% increase from baseline) occurred in 59.4% of the milrinone patients versus 15.6% of the placebo patients (OR 3.24, 95% CI 1.44–7.28; *p* = 0.001). Subgroup analyses considered age, sex, baseline mean arterial pressure, left ventricular ejection fraction, cardiac index, vasopressor dosages, serum lactate, and inferior vena cava diameter variation.

**Figure 3.**
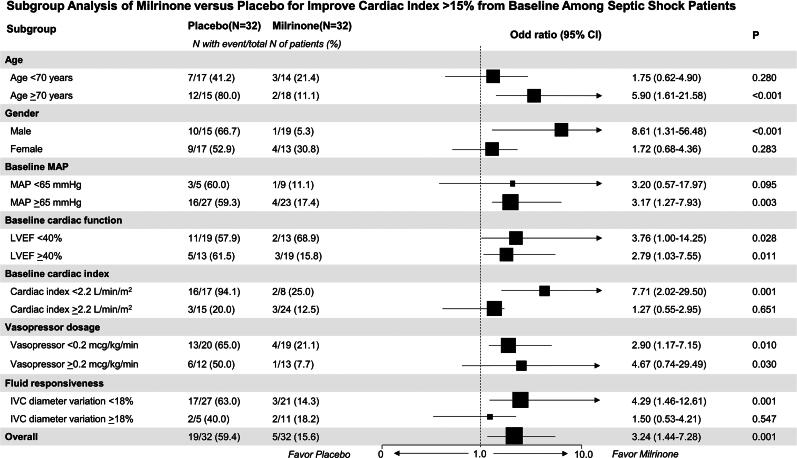
Forest Plot of subgroup analyses: stratification of patients with > 15% increase in cardiac index following study drug administration. Analysis based on eight patient characteristics. Square position: relative risk Square size: subgroup sample size Horizontal bars: 95% confidence intervals

Milrinone’s effect on cardiac index improvement was consistent across baseline left ventricular ejection fraction, vasopressor dosages, and serum lactate subgroups (Figure 3). However, milrinone showed significantly greater efficacy in patients ≥ 70 years (OR 5.90, 95% CI 1.61–21.58; *p* < 0.001), males (OR 8.61, 95% CI 1.31–56.48; *p* < 0.001), patients with a baseline mean arterial pressure ≥ 65 mmHg (OR 3.17, 95% CI 1.27–7.93; *p* = 0.003), patients with a baseline cardiac index < 2.2 L/min/m^2^ (OR 7.71, 95% CI 2.02–29.50; *p* = 0.001), and patients with an inferior vena cava diameter variation < 18% (OR 4.29, 95% CI 1.46–12.61; *p* = 0.001).

Milrinone did not significantly improve the cardiac index in patients < 70 years, females, patients with a baseline mean arterial pressure < 65 mmHg, patients with a baseline cardiac index ≥ 2.2 L/min/m^2^, or patients with an inferior vena cava diameter variation ≥ 18% (Figure 3).

## Discussion

This study investigated the effects of milrinone on persistent septic shock patients with persistent tissue hypoperfusion. Milrinone significantly increased cardiac output and the cardiac index at 6 h compared to placebo, with more milrinone patients showing *a* > 15% increase from baseline. However, no significant differences were observed in mortality, ICU length of stay, or organ support-free days. The incidence of adverse events and decreases in serum lactate levels were similar between the groups.

Limited research exists on the use of milrinone to improve cardiac output in refractory septic shock patients. Previous studies focused on pediatric septic shock [15,25], with only three addressing adult septic shock [16,26,27]. These studies demonstrated that milrinone can improve the cardiac index and, when combined with beta-blockers, enhance cardiac function, control heart rate, and potentially improve survival outcomes in patients with severe sepsis. Our results align with these studies, demonstrating milrinone’s ability to improve cardiac output in adult septic shock patients.

This study addresses a crucial knowledge gap in managing inadequate tissue perfusion in septic shock patients. By focusing on adult septic shock patients with poor tissue perfusion and utilizing bedside echocardiography, our findings demonstrate that adjunctive milrinone improves cardiac output in this population.

Although milrinone improved cardiac output in adult septic shock patients with inadequate tissue perfusion or impaired LV systolic function, it did not confer a mortality benefit. This finding aligns with a meta-analysis on milrinone use in critically ill adults with cardiac dysfunction [28] and a recent randomized controlled trial comparing milrinone to dobutamine in cardiogenic shock patients [14], both of which reported no mortality benefit.

Milrinone’s vasodilatory effects on arteries and veins, coupled with its inotropic properties, enhance ventricle–arterial coupling. These effects typically reduce pulmonary and systemic vascular resistance [29], potentially decreasing systemic blood pressure and increasing vasopressor requirements. In our study, slight increases in heart rate and vasopressor use were observed during milrinone administration, although these differences were not significant. Both parameters showed a declining trend throughout the study. Tachyarrhythmia, a common adverse effect of milrinone, occurred at the same incidence in the milrinone and placebo groups.

Our study suggested that the effect of milrinone on cardiac output may vary among specific patient subgroups. Significant cardiac index improvements were observed in patients > 70 years, males, and those with a baseline mean arterial pressure > 65 mmHg, a baseline cardiac index < 2.2 L/min/m^2^, and an inferior vena cava diameter variation < 18%. However, these findings warrant cautious interpretation due to the small size of the study population.

Patients with a mean arterial pressure greater than 65 mmHg may better tolerate the vasodilatory effects of milrinone. Increased blood pressure, associated with increased myocardial perfusion pressure, might enable a more effective myocardial response to milrinone’s inotropic effects. An inferior vena cava diameter variation of less than 18% may indicate fluid non-responsiveness, suggesting that inotropic treatment is potentially optimal for improving the cardiac index in patients with impaired left ventricular systolic function and a low cardiac index. Conversely, patients with an inferior vena cava diameter variation greater than 18% are more likely to exhibit fluid responsiveness and may benefit more from fluid therapy to improve the cardiac index.

Limited data exist on sex and age differences in the milrinone response. Physiological and hormonal variations between males and females could influence drug metabolism, efficacy, and side effects, potentially explaining response differences. Further research is needed to address age- and sex-specific responses to milrinone treatment.

The strength of this multicenter randomized controlled trial lies in its attempt to examine the efficacy of milrinone in adult septic shock patients with poor tissue perfusion. Hemodynamic changes were monitored *via* echocardiography, focusing on the left ventricular ejection fraction, inferior vena cava diameter variation, and cardiac output changes. A hands-on echocardiography workshop standardized procedures among operators, with inter- and intra-observer variabilities maintained below 15%. Additionally, strict blinding of the investigators, attending physicians, and medical staff minimized study bias.

However, our study has certain limitations. First, while the administration of the study drug was controlled, attending physicians could modify other vasoactive agents or fluid regimens, potentially influencing cardiac output changes. Second, lactate clearance was the sole tissue perfusion parameter assessed and can be affected by local tissue hypoxia or severe renal/hepatic dysfunction during septic shock. Third, the baseline imbalances in left ventricular ejection fraction and cardiac index also need to be acknowledged as a limitation, despite the lack of statistical significance. Even when baseline differences between groups are not statistically significant, they can still influence study outcomes, particularly when the variables are closely related to the outcome measure. Such imbalances could introduce bias and obscure the true treatment effect, especially when the variable in question is a strong predictor of the outcome. Furthermore, the study population was calculated based on cardiac output increase rather than the cardiac index due to the initial unavailability of beds capable of measuring patient body weight. However, before the study commenced, beds with this capability became available, allowing us to calculate the cardiac index as a secondary outcome. Last, the study may have been underpowered to assess mortality outcomes, as population calculations were not based on clinical outcomes such as ICU or hospital mortality. A larger multicenter study is needed to more precisely determine the mortality benefits of milrinone in septic shock patients.

## Conclusions

This study highlights the complexity of managing refractory septic shock with persistent tissue hypoperfusion. Although milrinone improved cardiac output, its impact on overall clinical outcomes, particularly mortality, remains uncertain. Potential adverse effects, including increased vasopressor requirements and tachyarrhythmias, must be carefully weighed against the benefits. Further research is necessary to elucidate the role of milrinone in this patient population and optimize its clinical application.

## Supplementary Material

Consort Milrinone.doc

## Data Availability

The datasets used and/or analysed during the current study are available from the corresponding author on reasonable request.
